# The Small-Molecule Enhancers of Autophagy AUTEN-67 and -99 Delay Ageing in *Drosophila* Striated Muscle Cells

**DOI:** 10.3390/ijms24098100

**Published:** 2023-04-30

**Authors:** Marcell Komlós, Janka Szinyákovics, Gergő Falcsik, Tímea Sigmond, Bálint Jezsó, Tibor Vellai, Tibor Kovács

**Affiliations:** 1 Department of Genetics, ELTE Eötvös Loránd University, 1117 Budapest, Hungary; komlos@gmail.com (M.K.); janka.szinyakovics@ttk.elte.hu (J.S.); falcsikgergo@student.elte.hu (G.F.); sigmond.timea@ttk.elte.hu (T.S.); 2 MTA-ELTE Genetic Research Group, 1117 Budapest, Hungary; 3 Department of Anatomy, Cell and Developmental Biology, ELTE Eötvös Loránd University, 1117 Budapest, Hungary; jezso.balint@ttk.elte.hu; 4 Institute of Enzymology, Research Center for Natural Sciences, Eötvös Loránd Research Network, 1117 Budapest, Hungary

**Keywords:** autophagy induction, muscle ageing, ageing, AUTEN-67, AUTEN-99, MTMR14, EDTP, *Drosophila*

## Abstract

Autophagy (cellular self-degradation) plays a major role in maintaining the functional integrity (homeostasis) of essentially all eukaryotic cells. During the process, superfluous and damaged cellular constituents are delivered into the lysosomal compartment for enzymatic degradation. In humans, age-related defects in autophagy have been linked to the incidence of various age-associated degenerative pathologies (e.g., cancer, neurodegenerative diseases, diabetes, tissue atrophy and fibrosis, and immune deficiency) and accelerated ageing. Muscle mass decreases at detectable levels already in middle-aged patients, and this change can increase up to 30–50% at age 80. AUTEN-67 and -99, two small-molecule enhancers of autophagy with cytoprotective and anti-ageing effects have been previously identified and initially characterized. These compounds can increase the life span in wild-type and neurodegenerative model strains of the fruit fly *Drosophila melanogaster*. Adult flies were treated with these AUTEN molecules via feeding. Fluorescence and electron microscopy and Western blotting were used to assess the level of autophagy and cellular senescence. Flying tests were used to measure the locomotor ability of the treated animals at different ages. In the current study, the effects of AUTEN-67 and -99 were observed on striated muscle cells using the *Drosophila* indirect flight muscle (IFM) as a model. The two molecules were capable of inducing autophagy in IFM cells, thereby lowering the accumulation of protein aggregates and damaged mitochondria, both characterizing muscle ageing. Furthermore, the two molecules significantly improved the flying ability of treated animals. AUTEN-67 and -99 decrease the rate at which striated muscle cells age. These results may have a significant medical relevance that could be further examined in mammalian models.

## 1. Introduction

Autophagy is an evolutionarily conserved, lysosome-dependent, self-degradation process of eukaryotic cells by which superfluous and damaged cellular constituents can be effectively eliminated from the cytoplasm [1,2,3,4,5]. This catabolic process provides energy and monomers for synthetic processes [6]. Thus, autophagy is essential for maintaining cellular homeostasis. The capacity of the autophagic process exhibits a gradual decline during the adult life span, thereby contributing to the development of various age-related degenerative diseases (such as cancer, diabetes, muscle atrophy, tissue fibrosis, diverse forms of neurodegenerative processes and immune deficiency) [7,8,9]. The pharmacological activation of autophagy could be a potent strategy in treating such human pathologies and extending a healthy life span. However, most of the autophagy-inducing compounds identified to date act upstream of the process and hence exert multiple unspecific effects. For example, rapamycin and Torin1 [10,11,12] activate autophagy by blocking the mTORC1 (mechanistic target of rapamycin kinase complex 1) complex, a major cellular inhibitor of autophagic degradation [13], which also affects several other cellular processes including protein synthesis, ribosome biogenesis and lipid biosynthesis [14,15]. Therefore, there is a growing interest in identifying novel drug candidates that can specifically enhance the autophagic process, thereby restoring its activity to basal levels at advanced ages.

Based on the mechanisms by which the autophagic cargo is delivered into lysosomes, three major types of autophagy can be distinguished: macroautophagy, chaperone-mediated autophagy and microautophagy [16,17,18,19,20,21,22]. Among these processes, macroautophagy is the most intensely characterized and best understood [23]. During macroautophagy (hereafter referred to as autophagy), a double isolation membrane structure called phagophore is formed around the cytoplasmic material destined to be degraded. When the growth of the isolation membrane is completed, a vesicle-like structure called an autophagosome is generated. The autophagosome then fuses with a lysosome to form an autolysosome, in which the enclosed cytoplasmic materials are degraded by acidic hydrolases [24]. The autophagic process is regulated by several specific protein complexes. Among them, the Vps34 (vacuole protein sorting 34) kinase-containing complex converts the substrate phosphatidylinositol (PI) lipid into phosphatidylinositol-3 phosphate (PI3P), which eventually designates membranes with different origins for the autophagic pathway [25]. The autophagic isolation membrane is generated from different intracellular membranes containing PI3P, to which other specific autophagy proteins (Atg—autophagy-related) are recruited [26,27]. One example of such an Atg protein is *Drosophila* Atg18, the fly ortholog of mammalian WIPI1/2 (WD repeat domain phosphoinositide-interacting protein 1/2). By binding to PI3P, Atg18 is capable of recruiting the Atg12-Atg5-Atg16 and Atg8/LC3B-containing (LC3B–1A/1B light chain 3B) complexes to the forming isolation membrane [16,28]. Hence, the Vps34 complex has a prominent role in autophagy control.

**Figure 1:**
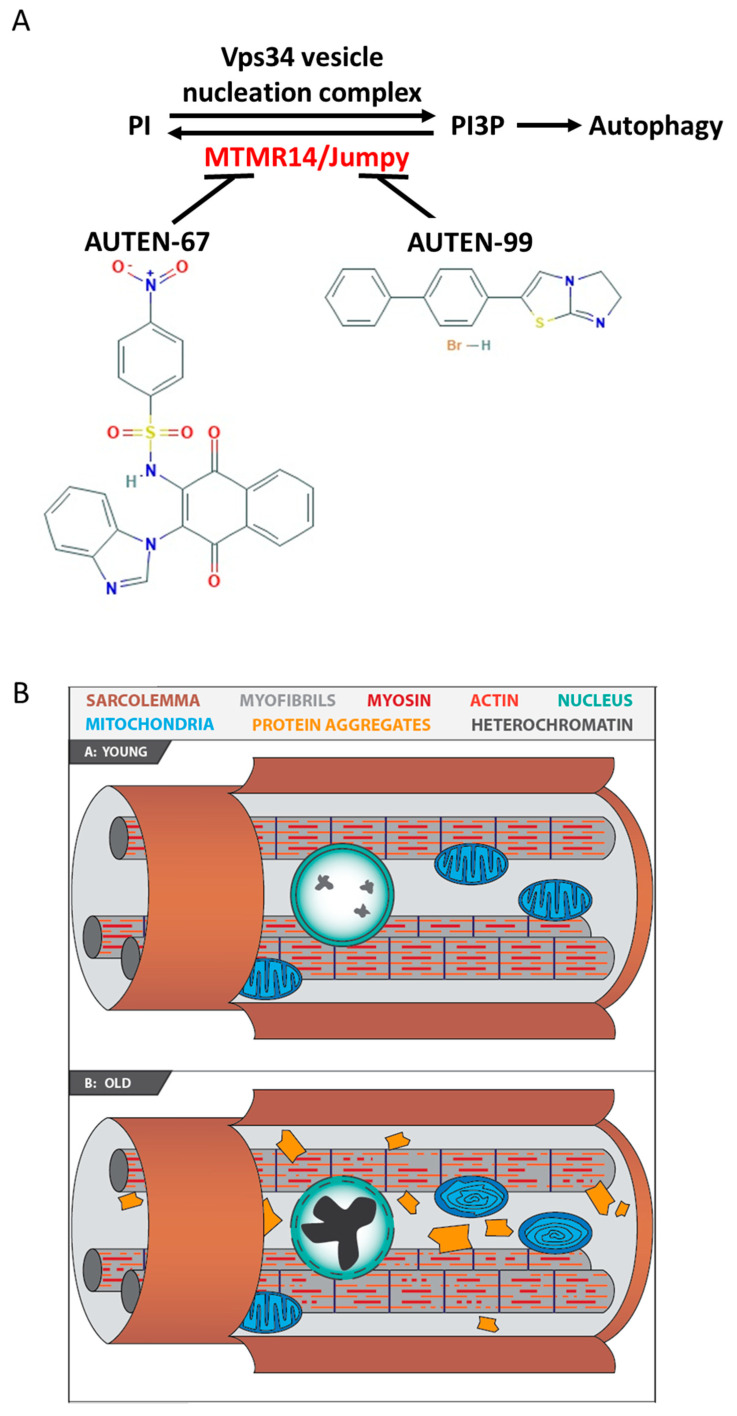
Mechanisms by which AUTEN-67 and -99 activate autophagy. (**A**) AUTEN-67 and -99 can induce autophagic degradation through interfering with MTMR14/EDTP. Converting PI into PI3P is an essential step for the formation of the autophagic isolation membrane. This process is catalyzed by the Vps34 kinase complex. In mammals, MTMR14/Jumpy antagonizes with the complex. MTMR14/Jumpy is a shared target of the two AUTEN molecules used in this study. EDTP, the *Drosophila* orthologue of MTMR14/Jumpy, can also be blocked by these AUTEN molecules. (**B**) Striated muscle from a young and an old animal. In the old specimen, protein aggregates (yellow) and damaged mitochondria containing abnormal internal lamellas (blue) accumulate, and there are nuclei with a highly condensed chromatin structure (green).

Mammalian MTMR14/Jumpy (myotubularin-related/Jumpy lipid phosphatase) and its *Drosophila* orthologue EDTP (egg-derived tyrosine phosphatase) antagonize with the Vps34 complex in controlling autophagic degradation [29,30,31]. Indeed, defects in MTMR14/Jumpy and EDTP functions lead to an enhanced activity of autophagy [29,32,33]. Previously, two autophagy-enhancing (AUTEN) small molecules were identified (Figure 1A), and it was found that they decrease the activity of MTMR14/Jumpy and EDTP in human cell lines and *Drosophila* larval tissues (fat body and brain), respectively [19,20]. AUTEN-67 also induces autophagy in primary neurons [34]. In *Drosophila*, this intervention could enhance the life span and improve motor functions in wild-type animals as well as in strains modeling Parkinson’s disease (PD) and Huntington’s disease (HD) [19,20]. Based on these data, AUTEN molecules may serve as promising drug candidates for the effective treatment of multiple neurodegenerative conditions. In the case of a drug candidate, potential side effects must be examined. Consistent with this statement, inactivating mutations in human MTMR-related phosphatases can cause the development of a muscle-related disorder called centronuclear myopathy (CNM) [35]. CNM is a group of congenital myopathies characterized by the presence of an abnormally large number of muscle fibers and the nuclei being arranged in rows in the central part of the fiber [36,37].

In this study, the effects of AUTEN-67 and -99 were analyzed on *Drosophila* striated muscle cells. As a model system, the indirect flight muscle (IFM) was selected, which is the largest and best-examined striated muscle tissue in this organism. Because the *Drosophila* striated muscle displays a strong functional similarity to mammalian skeletal muscles (regarding structure, metabolism as well as age-associated defects in neuro-muscular linkages), it is considered a tractable model for studying muscle ageing (Figure 1B) [38]. A decline in the capacity of autophagic during ageing is also a characteristic feature of these muscle systems [39]. In *Drosophila*, a functional decline in muscle tissue can be assessed by performing adequate flying and climbing assays [40,41]. The indirect flight muscle (IFM) is the largest and best-studied urinary striated muscle in Drosophila. The present study found that AUTEN-67 and -99 induce the autophagic process in the IFM, and improve flying ability in treated animals.

## 2. Results

### 2.1. AUTEN-67 and -99 Induce Autophagy in the Drosophila Striated Muscle

According to previous results, treatments of *Drosophila* with the AUTEN molecules (AUTEN-67 and -99) enhance the ability of animals to climb up on the wall of a glass vial [18,20]. In these earlier studies, however, enhanced autophagic activity upon AUTEN treatment was demonstrated only in the brain tissue of adult animals. It is reasonable to assume that improvement in moving ability can also result from enhanced autophagy in the muscle tissue. Hence, here, we studied the autophagic process in the IFM of control flies and animals treated by AUTEN-67 and -99 at different adult stages. AUTEN (which were dissolved in DMSO)-treated animals were compared with age-matched controls (exposed to DMSO only).

**Figure 2:**
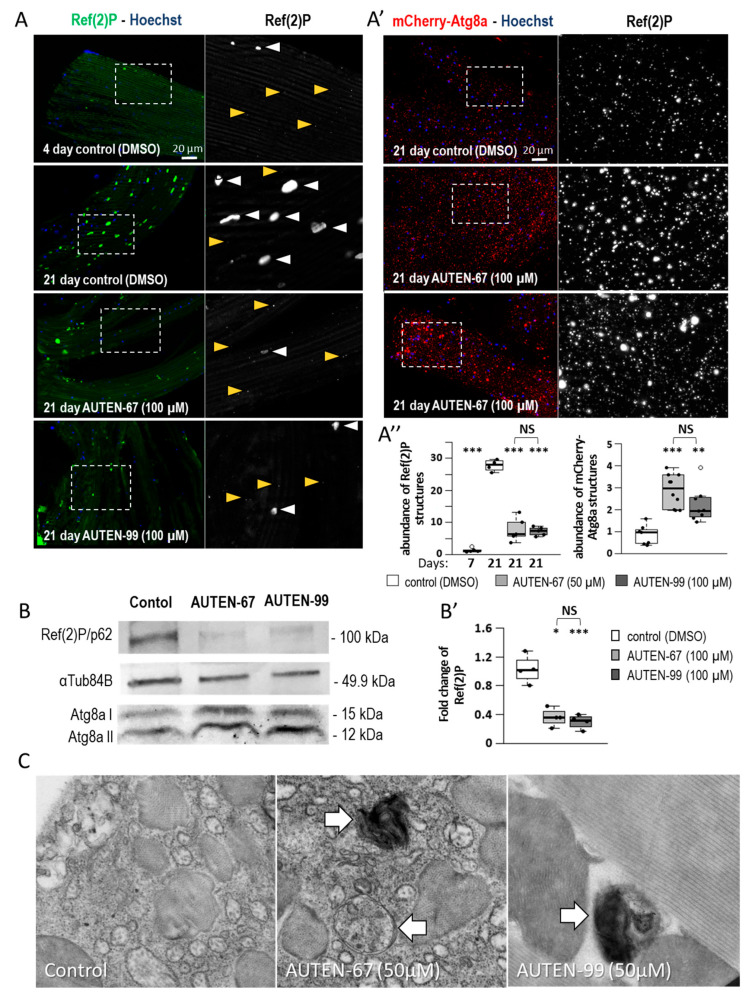
AUTEN-67 and 99 are capable of inducing autophagy in striated muscle. (**A**,**A″**) The amount of Ref(2)P/p62 serving as a substrate of autophagy is inversely related to the capacity of the process. AUTEN molecules decrease the amount of Ref(2)P-positive aggregates in the striated muscle of 21-day-old animals maintained at 29 °C. Yellow arrowheads indicate Ref(2)P-positive structures characterizing normal tissues, whereas white arrowheads point to Ref(2)P-positive aggregates accumulating with age (**A′**,**A″**). The amount of mCherry-Atg8a-positive structures increases in animals treated with an AUTEN molecule, as compared with the untreated control. Nuclei are indicated by blue coloring (Hoechst staining). In the right panels, the area outlined with a dotted line is enlarged (**A**,**A′**). (**B**,**B′**) Western blot analysis showing that the amount of soluble Ref(2)P is also lowered in samples isolated from the IFM of animals at age of adult day 21. Difference between Atg8a-I (soluble) and Atg8a-II (membrane-bound) levels lowers in response to AUTEN treatments. αTub84B was used as an internal control. In panels (**A′**,**B′**), bars represent ±S.D. *: *p* < 0.05; **: *p* < 0.01; ***: *p* < 0.001. For statistics, see Section 3. (**C**) Transmission electron microscopic images showing enhanced levels of autophagy in the IFM of animals exposed to an AUTEN molecule. Arrows show autophagic structures, autophagosomes and autolysosomes. Animals were maintained at 29 °C.

Mammalian p62/SQSTM1 (sequestrome 1), the single *Drosophila* orthologue of which is Ref(2)P (refractory to sigma P), serves as a substrate for autophagic degradation. Therefore, the level of p62/SQSTM1 inversely correlates with autophagic activity; the higher the intracellular level of the substrate, the lower the activity of the process [42]. To monitor autophagic activity, Ref(2)P levels were measured in the IFM at different adult stages, and we found that these levels increase as the animal ages (Figure 2A,A″). These results indicate that autophagic activity gradually lowers with age in the examined tissue. Similar data have been previously obtained from other *Drosophila* tissues, including the nervous system [30,43]. Next, Ref(2)P levels were compared in the IFM between control and AUTEN-67/99-treated animals. The results showed that fewer amounts of the substrate were detectable in the treated animals relative to the control at the same ages (Figure 2A), indicating that the molecules potently induce autophagy in these cells.

Damaged proteins are frequently marked with ubiquitin, and ubiquitinated protein aggregates are primarily degraded by autophagy (i.e., ubiquitinated proteins represent another substrate group for the autophagic breakdown) [42,44]. Thus, ubiquitin levels in the IFM during adulthood (at adult stages of 7 days—young and 21 days—aged) were also monitored. Ubiquitin levels also significantly increased in older animals relative to younger ones but decreased in flies treated with an AUTEN compound compared with untreated control at the same age (Figure S1A,B′). Together, these data suggest that the capacity of autophagy in the IFM endogenously declines during ageing, and it can be enhanced by AUTEN molecules.

LC3B is another tractable marker for monitoring autophagic activity. The *Drosophila* orthologue of LC3B is Atg8a [16]. Atg8a binds to the PE of the phagophore, the initial structure of autophagosome formation [45]. Therefore, the protein can be found in the forming autophagic vesicle, and, later, in the inner membrane of the matured autophagosome. Atg8a is also degraded by autolysosomal enzymes [46]. An mCherry-Atg8a reporter readily labels early (phagophores and autophagosomes) and late (autolysosomes) autophagic structures [47]. This study found that AUTEN treatments increase the amount of mCherry-Atg8a-positive structures in the IFM. (Figure 2A′,A″). The number of lysosomal components can also be measured by labeling cathepsin-L hydrolase with a specific antibody [48]. The increased amounts of cathepsin-L-positive structures were detected in the IFM of animals treated with an AUTEN molecule as compared to untreated control at the same age (Figure S1C,C′).

Fluorescence microscopy can directly reveal autophagic membranous structures, insoluble protein aggregates and abnormal organelles, while Western blotting is primarily suitable to detect the level of mainly soluble cytoplasmic proteins. Using the latter method, the amounts of soluble Ref(2)P and ubiquitinated proteins showed a significant decrease in 21-day-old animals treated with AUTEN molecules, as compared with the control at the same stage (Figure 2B,B′ and Figure S2A,A′). The amounts of Atg8a isoform (I and II)-positive structures were also examined. The soluble Atg8a-I is a 15 kDa large protein, while its lipidated form (termed PE-Atg8a or Atg8aII) is only 12 kDa in size [49]. Because Atg8a-II is also degraded by autophagy, the protein can also be considered an autophagic substrate. Upon autophagy activation, Atg8a-II levels increase. Thus, changes in autophagy activity can be inferred from the relative ratio of Atg8a-I and Atg8a-II proteins [49]. The current study found that the application of AUTEN-67 and AUTEN-99 each can decrease the ratio of Atg8a-I/Atg8a-II proteins compared to age-matched control (Figure 2B and Figure S2B).

The effect of these small molecules on autophagic activity was also studied at the ultrastructural level, and increased numbers of autophagosomes and autolysosomes were observed in the IFM samples exposed to AUTEN treatment (Figure 2C). Thus, AUTEN molecules are able to induce autophagy in the *Drosophila* IFM.

### 2.2. AUTEN Molecules Decrease the Amount of Damaged Mitochondria in the IFM

**Figure 3:**
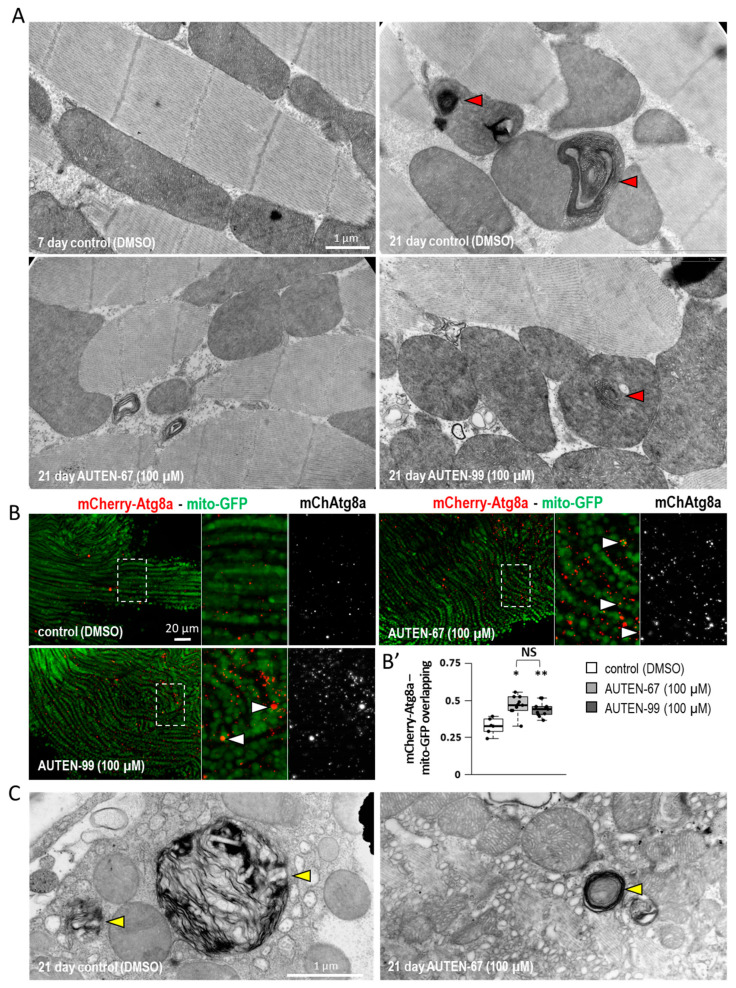
AUTEN small-molecule enhancers of autophagy delay ageing in *Drosophila* striated muscle. (**A**) Transmission electron microscopic images showing aberrant inner membrane structures of mitochondria (red arrowheads) in striated muscle cells of aged animals. The amount of abnormal structures was lowered in samples treated with an AUTEN molecule. (**B**,**B′**) The overlap between mitochondria (mito-GFP—green) and autophagic structures (mCherry-Atg8a—red) increases in samples treated with AUTEN-67 or -99 as compared with the untreated control. In the right panel, the area outlined by a dotted line is enlarged (**A**). In panel (**B′**), bars represent ±S.D. *: *p* < 0.05; **: *p* < 0.01. For statistics, see Section 3. (**C**) Electron microscopic analysis showing large multilamellar bodies (MLB—shown by yellow arrowheads) in the IFM of 21-day-old animals. The presence of these structures is indicative of an abnormal lysosomal breakdown. Animals of the same age display lower amounts of MLBs in response to AUTEN treatments. Animals were maintained at 29 °C.

Ageing in striated muscle can also be characterized by the accumulation of damaged, non-functional mitochondria, which are often wedged into protein aggregates [50,51]. Using transmission electron microscopy, the IFM was examined in young (7 days old) and old (21 days old) animals treated with an AUTEN molecule. This study found that samples from old flies contain higher amounts of damaged mitochondria than those derived from younger animals (Figure 3A). Furthermore, AUTEN treatment significantly decreased the number of mitochondria with abnormal inner lamellas as compared to untreated age-matched controls (Figure 3A). The elimination of damaged mitochondria by selective autophagy is called mitophagy [52]. For studying the mitophagic process, a colocalization assay was performed of mCherry-Atg8a- and mito-GFP-positive structures. The results showed that colocalization of these markers is enhanced in AUTEN-treated IFM samples relative to untreated age-matched controls (Figure 3B,B′). In samples from aged (21 days old) animals, multilamellar bodies (MLB) were observed, indicating a partial digestion of mitochondria and late endosomes containing intact inner membranes (Figure 3C) [53]. In the untreated IFM samples, significantly larger MBLs were detected as compared to treated ones (Figure 3C). The presence of enlarged MBL structures may refer to compromised lysosomal degradation [53]. It can be concluded that AUTEN molecules confer a beneficial effect on the elimination of damaged mitochondria.

### 2.3. AUTEN Molecules Improve Flying Ability in Drosophila

**Figure 4:**
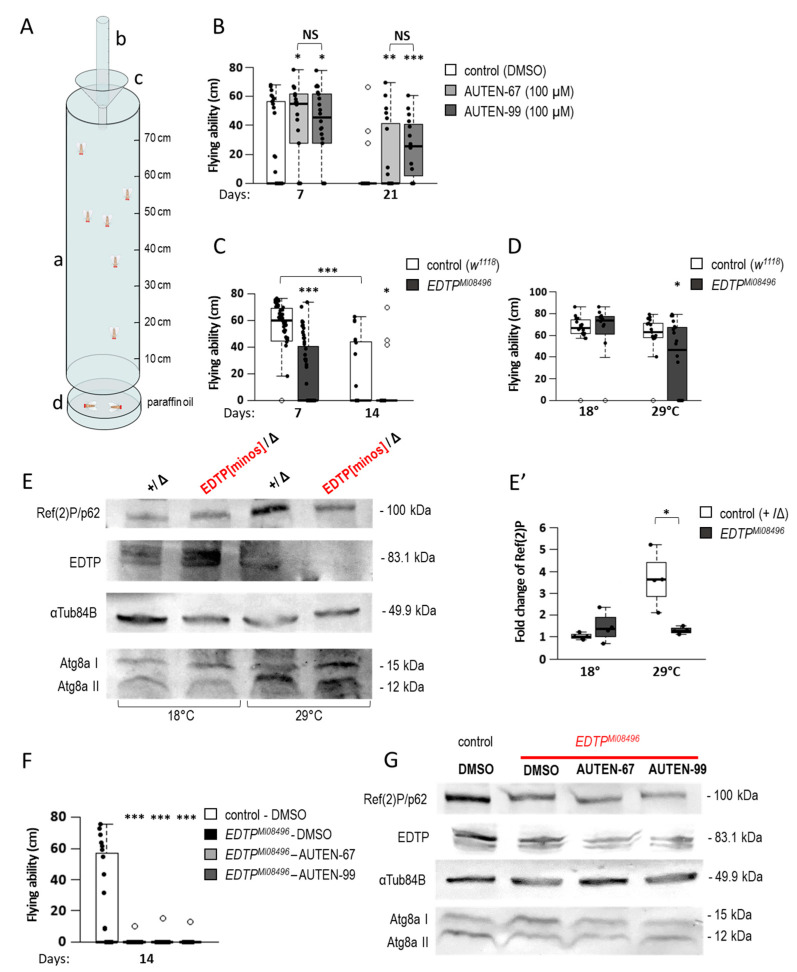
AUTEN-67 and -99 improve flying ability in *Drosophila*. (**A**) To perform a flying assay, a transparent, 80 cm high plexiglass measuring tube (a) was used. A paper covered by a transparent insect trap was placed on the inner wall of the tube. Glass vials containing animals were dropped in an inverted orientation into a drop tube on the top (b). The vial was stopped by a funnel (c) while animals kept falling into the measuring tube. Fitter animals flying towards the light got stuck to the paper covered by the insect trap close to the top of the tube, whereas old and unhealthy animals did the same close to the bottom of the tube. Animals unable to fly dropped into the paraffin oil (d) found at the bottom of the tube. Between the measurements, the insect trap was removed from the tube to determine the height at which they got stuck. Before the next measurement, animals were removed from the paper trap. The cleared trap was reinstalled inside the tube. (**B**) AUTEN treatments significantly improved the ability of both young (7 days) and aged (21 days) adults to fly. Animals were kept at 29 °C. (**C**) A hypomorphic allele of EDTP, EDTPMI08496, decreases the ability of 7 and 14 days old animals to fly. (**D**) EDTPMI08496 is a thermosensitive allele with a restrictive temperature of 29 °C. Development of animals occurred at a permissive temperature (18 °C) (in this way, potential developmental effects influencing flying ability were excluded); then, animals were transferred at 29 °C. Flying ability of mutant animals defective for EDTP function failed at the restrictive temperature (29 °C) only. Hence, defects in flying were not a consequence of lacking EDTP function during development. (**E**,**E′**) Proteins were extracted from animals treated as shown in panel (**D**). Western blot analysis reveals that amounts of EDTP lower only in *EDTP^MI08496^* mutants maintained at 29 °C. The amount of Ref(2)P also decreased in these animals (maintained under a restrictive condition), but not in those maintained at 18 °C (a permissive condition). Similarly, the ratio of Atg8a-I/Atg8a-II levels in EDTPMI08496 mutants relative to control changed at 29 °C only. (**F**) Flying ability of control (treated with DMSO) and AUTEN-treated *EDTP^MI08496^* animals at the adult age day 14. In the absence of EDTP function, AUTEN molecules cannot improve the ability of animals to fly. (**G**) Specificity of AUTEN-67 and -99 was also assessed by a Western blot analysis. Ref(2)P level decreased in EDTPMI08496 mutants, and this change cannot be influenced by an AUTEN treatment. Thus, these AUTEN molecules exert their effect on flying ability by inhibiting EDTP. In panels (**B**,**D**,**E′**,**F**), bars represent ±S.D. *: *p* < 0.05; **: *p* < 0.01; ***: *p* < 0.001. For statistics, see Section 3.

Next, the functional activity of striated muscle was tested in AUTEN-treated animals. The effect of AUTEN molecules on movement was assessed by using a flight test. In this method, selected animals are dropped into a long transparent tube, the wall of which is covered with an insect trap. In the tube, animals switch from falling to flying (flying toward the light) and get stuck in the trap. Fitter animals stick out at the top of the tube, while aged or abnormally flying flies stick out closer to the bottom of the tube. Less-flying animals fall into a paraffin oil collection container under the pipe (Figure 4A) [54]. The current study showed that AUTEN molecules increase the flying ability of both young and aged flies (Figure 4B). The effects of the thermosensitive hypomorphic mutant *EDTP^MI0849^* (hereafter referred to as *EDTP^MI^*) were also tested. *EDTP^MI^* mutant adults died significantly earlier at 29 °C than the control (Figure 4C), so the analysis could only be performed on animals aged 7 and 14 days old. As expected, *EDTP^MI^* mutants flew less than age-matched controls (Figure 4C). Thus, pharmacological and genetic inhibitions of EDTP led to opposing results. This implies that a developmental role of EDTP influences flying ability in adults. AUTEN treatments were performed only at adult stages, while the mutation affects EDTP function throughout the entire lifespan, including development. Therefore, flying assays on *EDTP^MI^* mutants were repeated in a specific way; animals were maintained under permissive conditions (at 18 °C) during development, then transferred to 29 °C (restrictive condition) throughout adulthood. A decline in flying ability was also detectable in mutant animals (Figure 4D). Under these conditions, further Western blot analysis confirmed that the amount of EDTP proteins in the *EDTP^MI^* genetic background does not decrease at 18 °C; in line with this finding, the amounts of the autophagic substrates Ref(2)P and Atg8a-II remained unchanged compared to the control kept under the same conditions (Figure 4E,E′ and Figure S3A). Under restrictive conditions, however, the amounts of EDTP and Ref(2) decreased significantly (Figure 4E,E′ and Figure S3A). Thus, decreased moving ability caused by EDTP deficiency is partially independent of the developmental role of EDTP. Finally, the specificity of AUTEN-67 or -99 to EDTP was tested in the muscle. After treatment with AUTEN-67 and -99, the flying abilities of *EDTP^MI^* mutants were measured. The flying ability of treated mutants was not changed as compared with untreated controls (Figure 4F). In this study, the autophagic activity was also observed in *EDTP^MI^* mutants treated with an AUTEN. Another Western blot analysis confirmed that EDTP protein levels were lowered in these mutants relative to the control genetic background (Figure 4G). Consistent with these data, the amount of Ref(2)P was also lower in the control and treated mutants (Figure 4G). So, treatment with an AUTEN cannot further increase autophagic activity in *EDTP^MI^* mutant animals. These results suggest that AUTEN-67 and -99 are specific to EDTP.

## 3. Discussion

AUTEN-67 and AUTEN-99 appear to act as specific inhibitors of MTMR proteins, and previous research uncovered AUTEN-type small-molecule enhancers of autophagy as potent agents in treating various neurodegenerative pathologies [18,19,20]. Mutational inactivation of MTMRs leads to muscle-related diseases in humans, raising the possibility that AUTENs may also contribute side effects in the muscle tissue [35,54]. In the present study, the effects of AUTEN-67 and -99 were examined on the age-related functionality of *Drosophila* striated muscle cells and excluded this negative effect.

Using fluorescent and electron microscopy, as well as Western blot analyses, this study showed that autophagy is activated in the IFM of animals treated with an AUTEN molecule (Figure 2 and Figure S1). AUTEN-67 and -99 had similar effects on autophagy activation. During ageing, unfunctional mitochondria accumulate in the muscle tissue and may significantly interfere with its working capacity [55]. Moreover, damaged mitochondria can produce reactive oxygen species serving as cellular toxins [56]. The accumulation of tangled mitochondria can also lead to mitochondrial myopathies, which can lead to weakness, muscular dysfunction and a host of other symptoms [57]. Vesicle nucleation promoted by AUTEN molecules was likely to contribute to the degradation of damaged mitochondria. This is supported by the result that upon treatment with an AUTEN molecule, autophagy structures significantly overlapped with labeled mitochondria (Figure 3B,B′), and that in the IFM of aged animals, there were fewer damaged mitochondria containing abnormal lamellas than in controls (Figure 3).

The current study also measured the effect of AUTEN molecules on muscle function by using a flying assay. Consistent with previously published data from climbing assays [18,20], AUTEN treatments improved the ability of *Drosophila* to fly (Figure 4B). In contrast to this pharmacological inhibition, flying ability was found to be significantly worse in *EDTP* hypomorphic mutant animals compared to wild-type ones (Figure 4C). It is worth noting that this change was not influenced by development because EDTP functions were intact until the onset of adulthood (hypomorphic *EDTP^MI08496^* mutants were maintained at a permissive temperature during development) (Figure 4C,D). Thus, it can be concluded that pharmacological (AUTEN-mediated) inhibition of EDTP reduces, but does not eliminate, the function of the protein. This can moderately induce autophagic activity; that is, AUTEN treatment can restore the process to basal (physiological) levels.

Differences in results between pharmacological and genetic inhibitions of EDTP show that these AUTEN molecules are not exclusively specific to EDTP; rather, they may also interfere with other myotubularin-related lipid phosphatases [29,35]. This prompted us to monitor the specificity of AUTENs in the IFM. Both AUTEN-67 and -99 failed to improve flying ability in EDTP-defective mutant animals and consistently did not increase autophagic and flying activities in the IFM of *EDTP^MI08496^* mutant animals (Figure 4F,G). Thus, these small-molecule enhancers of autophagy appear to specifically affect EDTP in the IFM of adult animals.

Although the *Drosophila* IFM is a tractable model for studying muscle ageing, the system possesses several limitations [58]. For example, muscle regeneration characterizing mammalian organisms, as well as the age-related loss of muscle mass, cannot be examined in the fruit fly [59,60,61,62]. As in the nervous system, the IFM has been shown to increase EDTP expression with age [30,31,63]. If the age-dependent increase in EDTP expression causes harmful protein dephosphorylation in muscle, it may be important to investigate the effect of AUTENs on phosphatase activity in IFM ageing in a future study. Hence, further studies should analyze whether AUTENs can also delay muscle ageing and influence the above processes in mammalian systems. If these compounds positively affect muscle functions at advanced ages, they will serve as potent drug candidates for inhibiting the progression of diverse age-associated symptoms.

We previously demonstrated that in *Drosophila*, AUTEN-67 and -99 are capable of inducing the autophagic process in neural tissue [18,20], and can also enhance the capacity of the process in larval fat body cells [19,20]. In this study, evidence was provided that these small molecules are able to induce autophagy in IFM cells, thereby delaying ageing in this tissue. In the future, it is worth assaying the autophagy-inducing effect of AUTEN molecules in other tissues and disease models.

## 4. Materials and Methods

### 4.1. Drosophila Strains

*Drosophila* strain stocks were maintained on standard cornmeal–sugar–agar medium at 25 °C, and experiments were carried out at 28 °C unless noted. Strains were obtained from the Bloomington Drosophila Stock Center (BDSC) or kindly provided by other researchers.

The following alleles were used (strains):

*w^1118^* (BDSC 5905);
*EDTP^MI08496^* (BDSC 44782);
*Df(2R)BSC161* (BDSC 9596);
*y*; *3xmCherry-Atg8a^4-14^* (II) (as a gift of Gábor Juhász, ELTE Eötvös Loránd Universty, Budapest, Hungary [55] (cit);
w*; *P{w^+mC^ = sqh-EYFP-Mito}* (III) (BDSC 7194).

### 4.2. AUTEN Treatment

The small molecules AUTEN-67 (AOBIOUS, AOB33340) and AUTEN-99 (AOBIOUS, AOB8904) were dissolved in DMSO [19,20]. Control animals were treated with DMSO only. Final concentrations (50 µM AUTEN-67 and 100 µM AUTEN-99) were prepared in heated yeast suspension, and treatments were performed via feeding. During the treatment regiments, animals were transferred into fresh vials every 2 days.

### 4.3. Western Blotting

Protein samples (~1 mg) were isolated from the dorsal part of the thorax. Western blotting was carried out by using an SDS-containing gradient acrylamide gel, nitrocellulose blotting membrane, and TBST washing solution. In addition, 3% milk powder solved in TBST was used. Proteins were labeled at 4 °C overnight by the following primary antibodies: anti-ubiquitin (mouse, 1:500, Merck, Rahway, NJ, USA, ST1200), anti-Atg8a (rabbit, 1:2000 [64,65]), anti-p62-vel (rabbit, 1:2000 [56]), anti-α-Tub84B (mouse, 1:2500, Sigma, St. Louis, MO, USA, T6199) and anti-EDTP (rat, 1:500 [19]). The following secondary antibodies were used (at room temperature for an hour): anti-rabbit IgG alkaline phosphatase (1:1000, Sigma, A3687), anti-mouse IgG alkaline phosphatase (1:1000, Sigma, A8438), and anti-rat IgG alkaline phosphatase (1:1000, Sigma, A8438). Primary and secondary antibodies were washed out 3 times for 10 min in TBST, and finally, membranes were incubated in an AP buffer. NBT/Bcip (Sigma, 72091) was used for recording the antibody staining, and NBT/Bcip was dissolved in an AP buffer (1:50).

### 4.4. Immunohistochemistry

Dissected IFM samples were fixed in 4% formaldehyde (diluted in PBS) at room temperature for 30 min. Then, tissues were washed for 4 × 5 min in TBSX (PBS with 2% Triton-X) and incubated in 5% FBS serum for 30 min. The following primary antibodies were used: anti-p62 (rabbit, 1:200 [56]), anti-ubiquitin (mouse, 1:500, Merck, ST1200) and anti-cathepsin-L 1:200 (rabbit, Abcam, Cambridge, UK, ab58991). On the second day, samples were washed for 4 × 5 min and blocked for 30 min as described above. Secondary antibody labeling was then carried out at 4 °C: anti-mouse Texas Red (Life Technologies, Carlsbad, CA, USA, T862), anti-mouse 1:500 Alexa Fluor 488 (Life Technologies, A1108), anti-rabbit 1:500 Texas Red (Life Technologies, T2767) and anti-rabbit 1:500 Alexa Fluor 488 (Life Technologies, A11008). On the third day, samples were washed 3× for 20 min in PBST, and 1 × 20 in PBS. Nuclei were stained with 50 µM Hoechst (Molecular Probes, Eugene, OR, USA, H-1399).

### 4.5. Fluorescence Microscopy

Fluorescent imaging was carried out on a Zeiss Axioimager M2 fluorescent microscope, equipped with ApoTome.2 semi-confocal uptakes, Colibri 7 LED lights, an Axiocam 503 monochrome camera and a ZEN 2.3, blue edition software. EC Plan-NeoFluar 10 × 0.3 NA and Plan-NeoFluar 40 × 0.75 objectives were used.

### 4.6. Electron Microscopy

Ultrastructural analysis of the IFM samples was performed according to [66,67]. Durcupan/Fluca was used for embedding (Sigma-Aldrich, 44610-1EA). Images were recorded by a JEOL, JEM-1011 transmission microscope (ELTE Eötvös Loránd University, Budapest), operated at 80 kV accelerating voltage, equipped with a 11-megapixel Morada side-mounted CCD camera and iTEM software (Olympus, Tokyo, Japan).

### 4.7. Flying Assays

Animals were anesthetized with CO_2_, and rested for 1 h before the flying assay. Vials containing the flies were dropped into a 25 cm long drop tube, the bottom of which was narrowed with a funnel. Animals continued to fall through this opening. The measuring tube was 80 cm long; a paper covered by a transparent insect trap was placed on the inner wall of this tube (Figure 3A). Falling animals try to switch to flight and flee towards the light, which is the side of the tube because of the transparent paper. Fitter animals stick closer to the top of the tube, while old animals stick closer to the bottom of the tube or fall into a container that contains paraffin oil below the tube. To determine flying ability, the height at which they got stuck (distance from the bottom of the funnel) was recorded for each animal. Trapped animals were removed from the paper and oil before the next measurement [68].

### 4.8. Evaluation and Statistics

For the statistical analysis of flying measurement, Western blot and fluorescence microscopy, R Studio (Version 3.4.3), were used. The distribution of samples (normal or not) was tested with a Lilliefors test. If it was normal, the F-test was performed to compare variances. In case variances were equal, a two-sample *t*-test was used; otherwise, a *t*-test for unequal variances was applied. In the case of non-normal distribution, a Mann–Whitney U-test was performed. On the plot, the boxes represent the most typical 50% of the samples, the lines indicate the median and the upper and lower whiskers show the remaining 25–25% of the samples. Circles mark outliers. *p* < 0.05 *; *p* < 0.01 **; *p* < 0.001 ***.

## 5. Conclusions

These results show that both AUTEN-67 and AUTEN-99 can increase autophagy in the striated muscles of animals and thus contribute to the improvement of movement function. The drug treatments were successful in reducing morphological changes in the aged muscles. These data suggest that AUTEN molecules may be promising therapeutic molecules that may have positive effects on muscle tissue in addition to treating neurodegenerative disorders. However, the relevance of the current results is necessary to investigate in the future in vertebrate models as well as in myopathy disease models.

## Data Availability

The datasets used and/or analyzed during the current study are available from the corresponding author upon reasonable request.

## Supplementary Materials

The following supporting information can be downloaded at: https://www.mdpi.com/article/10.3390/ijms24098100/s1.
