# Supplementary material for: The Small-Molecule Enhancers of Autophagy AUTEN-67 and -99 Delay Ageing in Drosophila Striated Muscle Cells
Source: Int J Mol Sci. 2023 Apr 30;24(9):8100. doi: 10.3390/ijms24098100 (PMC10179358; doi:10.3390/ijms24098100)
Supplement: Supplementary file 1 [file ijms-24-08100-s001.zip › ijms-2348768-supplementary.pdf]

Supplementary figure legends

S.Fig1

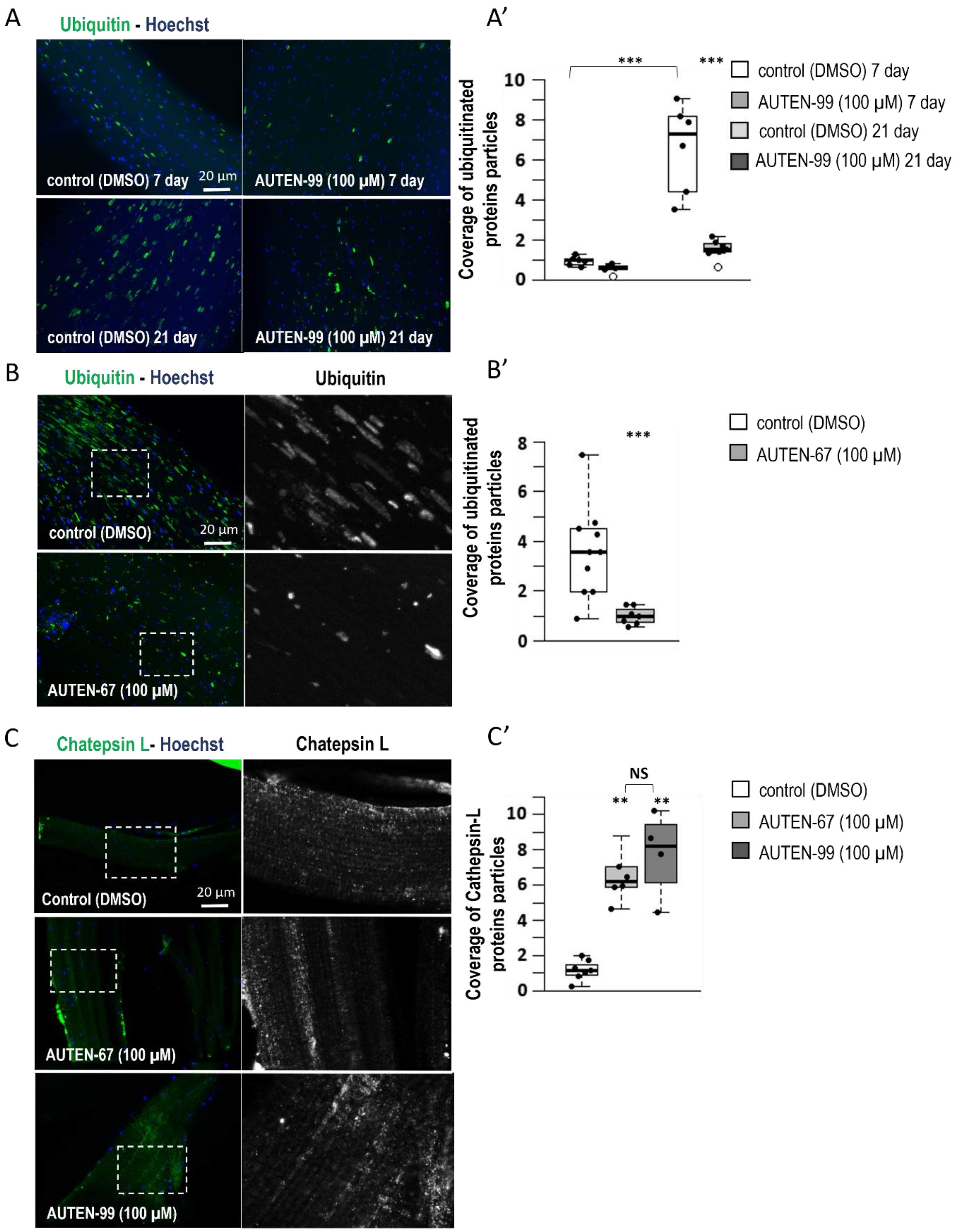

Figure S1. AUTENs trigger autophagy in Drosophila indirect flying muscle (IFM) (**A-B**) AUTEN-99 – AUTEN-67 enhances the degradation of ubiquitin aggregates at different ages.

Ubiquitin was labeled by anti-ubiquitin (green), and nuclei were stained with Hoechst (blue). (C) Cathepsin-L is a lysosomal enzyme. Thus, anti-Cathepsin-L (green) represents the IMF's lysosomal structures (lysosomes and autolysosomes). Both AUTEN-67 and -99 elevate the abundance of Cathepsin-L-positive structures. Hoechst (blue) staining labels the nuclei. (A’,B’,C’) On the plots black puncta label single measure points, the boxes represent the most typical 50% of the samples, the line indicates the median, upper and lower whiskers show remaining 25%-25% of the samples. Circles mark outliers. *: $P<0.05$; **: $P<0.01$; ***: $P<0.001$ at each comparison with control DMSO treatment. For statistics, see the Materials and Methods.

S.Fig2

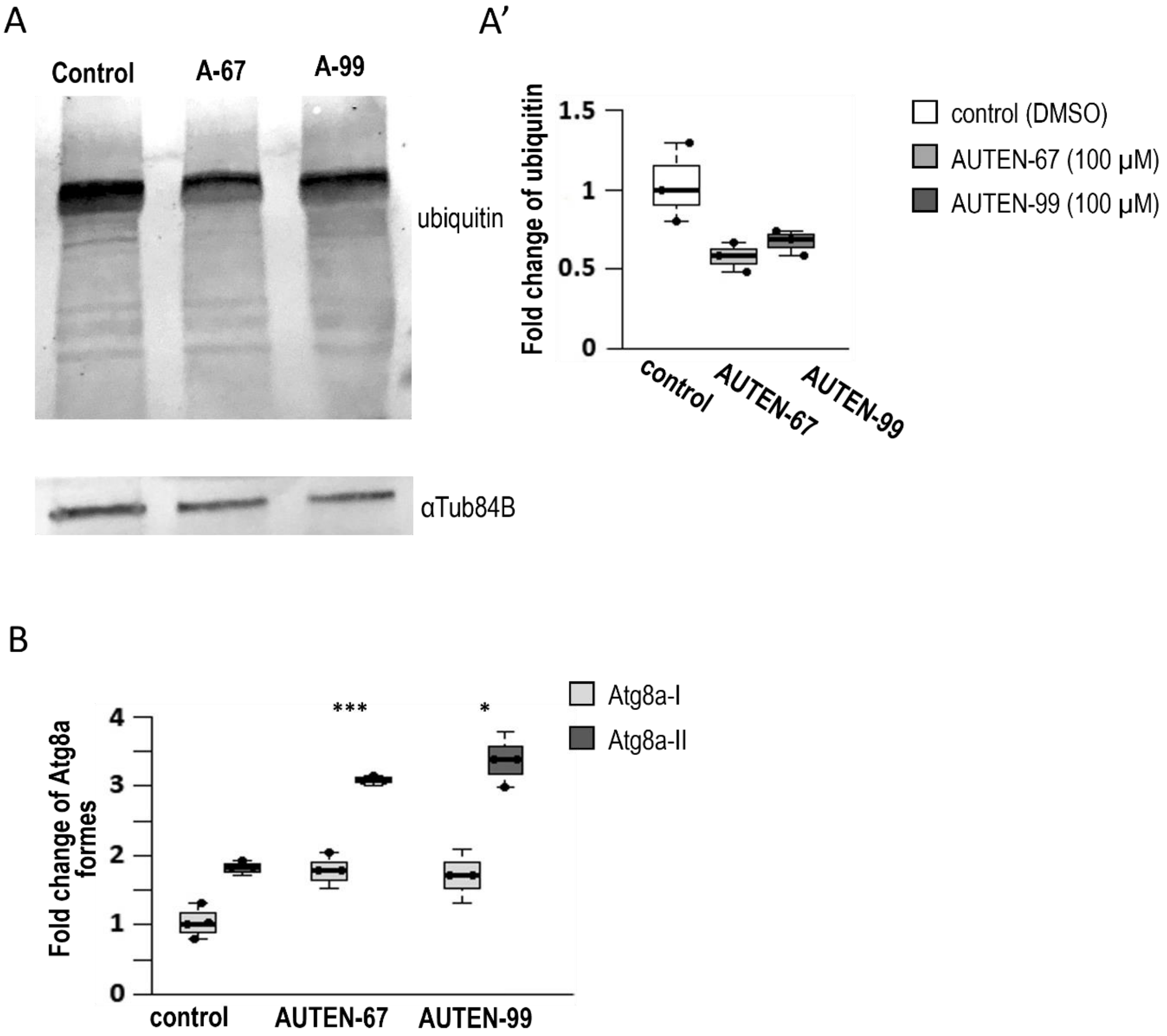

Figure S2. AUTENs induce lysosomal protein degradation (A-A’) AUTEN-67 and -99 enhance the degradation of ubiquitinated proteins. During Western blot measurements, ubiquitin was labeled with a septic antibody, and anti-αTub84B was used as an internal control. (B) Boxplot diagrams show the variation of soluble (Atg8a-I) and lipidated (Atg8a-II) Atg8a

levels in AUTEN-treated and control (DMSO) samples. The Western blot image can be seen in Fig.2.B. All protein samples were isolated from IFM. On the plots black puncta label single measure points, the boxes represent the most typical 50% of the samples, the line indicates the median, upper and lower whiskers show remaining 25%-25% of the samples. Circles mark outliers. *: $P<0.05$; **: $P<0.01$; ***: $P<0.001$ at each comparison with control DMSO treatment. For statistics, see the Materials and Methods.

S.Fig3

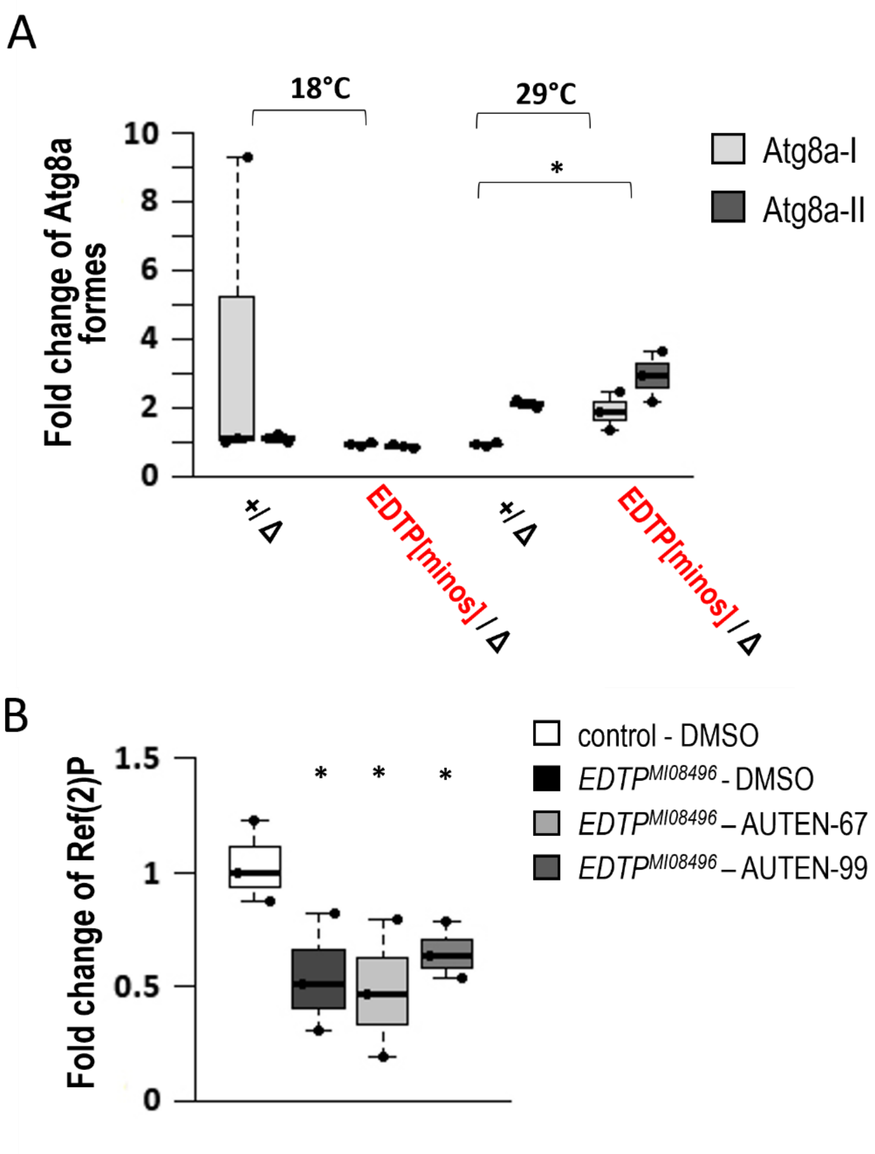

Figure S3. AUTENs induce autophagy by the specific repression of EDTP (A) A hypomorphic allele of EDTP, EDTP^{MI08496} was tested for thermosensitivity and we compared the protein levels of Atg8a-I and Atg8a-II in mutant and wildtype conditions on restrictive (29°C) end permissive (18°C) conditions. The Western blot image can be found in Fig.4.E. **(B)** Ref(2)P level decreases in $EDTP^{MI08496}$ mutants, and this change cannot be influenced by an AUTEN treatment. Thus, these AUTEN molecules exert their effect on flying ability through inhibiting EDTP. The Western blot image can be found in Fig.4.G. All protein samples were isolated from IFM. On the box-plots black puncta label single measure points, the boxes represent the most typical 50% of the samples, the line indicates the median, upper and lower whiskers show remaining 25%-25% of the samples. Circles mark outliers. *: $P<0.05$; **: $P<0.01$; ***: $P<0.001$ at each comparison with control DMSO treatment. For statistics, see the Materials and Methods.
